# Size Matters: The CAG Repeat Length of the Androgen Receptor Gene, Testosterone, and Male Adolescent Depression Severity

**DOI:** 10.3389/fpsyt.2021.732759

**Published:** 2021-10-20

**Authors:** Raphael Hirtz, Lars Libuda, Anke Hinney, Manuel Föcker, Judith Bühlmeier, Paul-Martin Holterhus, Alexandra Kulle, Cordula Kiewert, Johannes Hebebrand, Corinna Grasemann

**Affiliations:** ^1^Division of Pediatric Endocrinology and Diabetology, Department of Pediatrics II, University Hospital Essen, University of Duisburg-Essen, Essen, Germany; ^2^Department of Child and Adolescent Psychiatry and Psychotherapy, University Hospital Essen, University of Duisburg-Essen, Essen, Germany; ^3^Faculty of Natural Sciences, Institute of Nutrition, Consumption and Health, University Paderborn, Paderborn, Germany; ^4^Department of Child and Adolescent Psychiatry, University Hospital Münster, Münster, Germany; ^5^Department of Paediatrics I, Paediatric Endocrinology and Diabetes, University Medical Center Schleswig-Holstein (UKSH), Campus Kiel, Christian-Albrechts-University, Kiel, Germany; ^6^Department of Pediatrics, St. Josef-Hospital, Center for Rare Diseases (CeSER), Ruhr-University Bochum, Bochum, Germany

**Keywords:** depression, adolescents, testosterone, androgen receptor, CAG repeat length

## Abstract

There is a distinct increase in the prevalence of depression with the onset of puberty. The role of peripubertal testosterone levels in boys in this context is insufficiently understood and may be modulated by a functional polymorphism of the androgen receptor gene (AR), a variable number of CAG repeats. Moreover, there is preliminary evidence that the relationship between testosterone, CAG repeat length, and the severity of depressive symptoms may differ between subclinical and overt depression, but this has neither been studied in a clinical sample of adolescents with depression nor compared between subclinical and overt depression in an adequately powered study. To investigate the relationship between free testosterone, CAG repeat length of the AR, depression status (subclinical vs. overt), and the severity of depressive symptoms, 118 boys treated as in- or daycare patients at a single psychiatric hospital were studied. Of these, 73 boys had at least mild depressive symptoms according to the Beck Depression Inventory-II (BDI-II > 13). Higher-order moderation analysis in the multiple regression framework revealed a constant relationship between free testosterone and depression severity irrespective of the number of CAG repeats in adolescents with a BDI-II score ≤ 13. In adolescents with a BDI-II score > 13, however, there was a significant negative relationship between free testosterone and BDI-II score in patients with <19 CAG repeats and a significant positive relationship regarding free testosterone and BDI-II score in those with more than 28 CAG repeats, even when considering important covariates. These results suggest that the effects of testosterone on mood in male adolescents with depression depend on the genetic make-up of the AR as well as on depression status. This complex relationship should be considered by future studies addressing mental health issues against an endocrine background and may, moreover, contribute to tailored treatment concepts in psychiatric medicine, especially in adults.

## Introduction

There is a distinct increase in the prevalence of depression with the onset of puberty (1), especially in those adolescents who experience puberty earlier than their peers (2) or rush through puberty (3). It has been speculated that this observation is related to the fundamental and rapid hormonal changes triggered by the onset of puberty (3). However, in boys, Duke et al. (4) did not identify a significant relationship between depressive symptoms and testosterone levels in a systematic review of community studies, but these studies included only a small number of clinically depressed children and adolescents. Considering that clinical studies consistently report larger effect sizes regarding outcome measures in mood disorders than community studies (5), the aspect of depression severity warrants consideration in the relationship between testosterone and depression.

It is feasible that an influence of testosterone on depressive symptoms in adolescents is moderated by covariates, including the responsiveness of its receptor. The effect of testosterone on androgen receptor-related signaling has been shown to inversely depend on a polymorphic CAG repeat of variable length in exon 1 of the *AR* such that fewer CAG repeats are related to a more pronounced effect of testosterone on gene transcription *in vitro* (6) as well *in vivo* (7). These findings also translate into clinical research by the observation that the CAG repeat length of the *AR* (subsequently: CAG-RL) is related to Kennedy's disease, an inherited neuromuscular disorder, occurring if the number of CAG repeats exceeds a threshold of (likely) at least 39 copies (8). However, also within the normal range of CAG copy numbers, the absolute number of CAG repeats has been related to cognitive functioning (9), confidence and competitiveness (10), as well as psychiatric disorders, including mood disorders. Regarding the latter, the CAG-RL has been shown to affect the relationship between testosterone and depression severity in adults (11, 12) as well as adolescents (13): While in neither of these studies, a bivariate correlation between testosterone levels and depression severity was found, a significant interaction between testosterone, depression severity, and CAG-RL was observed (11, 13). Interestingly, studies in adolescents and adults yielded conflicting findings regarding this interaction. In a community sample of 301 adolescents, Vermeersch et al. (13) found that fewer CAG repeats (≤20) and higher levels of testosterone were related to more severe but mostly subclinical depression when compared to longer CAG repeats (>23). In contrast, two observational studies of 110 (11) and 201 (12) adults with clinically significant depressive symptoms found the opposite effect, fewer CAG repeats and higher levels of testosterone were associated with less depression than longer CAG repeats. Considering these inconsistent findings, the strength but also direction of the relationship between testosterone, CAG-RL, and depression may depend not only on the severity of depression, as mentioned earlier, but also on developmental aspects of endocrine and mental functioning.

In addition to the potential moderating effect of the CAG-RL on the effects of testosterone in depression, adrenal steroids, which have been related to male and female adolescent mood disorders, should likewise be considered as potential confounders (14, 15).

Against this background, the present study aimed to investigate the relationship between testosterone, CAG-RL, and depression severity in a clinical sample of male adolescents with and without depression to study the impact of depression status. Further, the study was also intended to derive conclusions regarding the role of developmental aspects on this relationship by a comparison to findings in adults. All analyses were conducted considering important covariates known to affect testosterone levels, including adrenal steroids. The following hypotheses were tested:

*H*_1_: There is an interaction between testosterone and CAG-RL concerning depression severity.
*H*_2_: The interaction between testosterone and CAG-RL concerning depression severity is modulated by depression status (subclinical vs. overt depression).

## Methods

### Study Design and Participants

Data of the present study were derived from the baseline assessments of a two-armed parallel-group, double-blind RCT which investigated the effect of vitamin D deficiency [25(OH) vitamin D ≤ 12 ng/ml (equivalent to ≤ 30 nmol/l); DRKS00009758] on depressive symptoms in psychiatric in- or daycare patients treated at the Department of Child and Adolescent Psychiatry, Psychosomatics and Psychotherapy Essen (LVR-Klinikum Essen), Germany (16). Additionally, we used data from the “Nutrition and Mental Health” study, a cross-sectional study focusing on the relationship between nutrition and mental disorders. Both studies followed the same protocol and were conducted in accordance with the Declaration of Helsinki and approved by the local Ethics Committee (No. 15-6363-BO).

Patients were eligible for inclusion if they were aged 11–18.9 years. Exclusion criteria were a concurrent diagnosis of severe somatic disease and/or intellectual disability. For the present study, we considered all male patients with no missing information on any of the variables of interest. Moreover, patients with a BMI below 5th percentile [< -1.64 standard deviations (SD)] and/or a diagnosis of anorexia nervosa were excluded from analysis due to effects of starvation and malnutrition on gonadal functioning (17).

### Questionnaires

The BDI-II is a self-reported questionnaire that records the severity of depressive symptoms according to DSM-IV diagnostic criteria for major depressive disorder (MDD) over the past 2 weeks by 21 items with excellent reliability and a high agreement between self-reported and clinician assessed depression (18). Total scores between 0 and 13 indicate no, between 14 and 19 mild, between 20 and 28 moderate, and above 28 severe clinically significant depressive symptoms (19). Patients with a total BDI-II score > 13 were classified as depressed.

Psychiatric diagnoses were established *via* the semi-structured interview “Schedule for Affective Disorders and Schizophrenia for School-Aged Children–Present and Lifetime Version” (K-SADS-PL) according to DSM-IV (20) or clinical assessment according to ICD-10 when no K-SADS-PL was performed.

Additionally, covariates known to affect testosterone levels, including the intake of psychotropic medication and health-related behavior such as smoking were recorded on admission.

### Anthropometric Measures

Patients were subjected to a physical examination upon admission, including assessment of body height and body weight. Height was determined in an upright posture to the nearest 0.1 cm using a wall-mounted stadiometer. Bodyweight was measured in underwear by an electronic scale to the nearest 0.1 kg. BMI was determined by the ratio of weight in kg and the height in meters squared (kg/m^2^). To consider the effect of age, BMI was z-transformed according to percentile charts for German children and adolescents (21) [RefCurv Version 0.4.4, https://refcurv.com (22)].

As almost 20% of patients refused Tanner staging, a prepubertal status was defined by a testosterone level below 1.0 nmol/l (23).

### Laboratory Studies

Blood samples were obtained from an antecubital vein in monovettes (Sarstedt, Germany) in the early morning before 8 am after an overnight fast and transferred within 1 h after sampling to the laboratory of the University Hospital Essen for analyses. Serum and plasma aliquots were stored at −80°C until testosterone and cortisol were determined by liquid-chromatography tandem mass spectrometry [LC-MS/MS; (23, 24)]. In brief, the stored sample aliquots, aliquots of calibrator and controls with a volume of 0.1 mL were combined with the internal standard mixture to monitor recovery. All samples were extracted using Oasis MAX SPE system Plates (Waters, Milford, MA, USA). LC-MS/MS was performed using a Waters Quattro Premier/Xe triple-quadrupole mass spectrometer connected to a Waters Acquity (Waters, Milford, MA, USA; Table 1 for details on assays).

**Table 1:** Assays and their performance characteristics.

**Parameter**	**Assay system**	**Assay type**	**Intra-assay variation**	**Total assay variation**	**Detection range**
Albumin	Siemens^*1^ ADVIA	BCG	<1.1%	<1.8%	10–60 g/l
Androstendione	Siemens Immulite 2000	CLIA	<15.1%	<17.8%	1.04–35 nmol/l
DHEA-S	Siemens Immulite 2000	CLIA	<9.8%	<13%	0.41–27 μmol/L
SHBG	Siemens Immulite 2000	CLIA	<5.3%	<6.6%	up to 180 nmol/l
25(OH)-vitamin D	Siemens ADVIA	CLIA	<5.3%	<11.9%	10.5–375 nmol/l
CRP	Abbott^*2^ Architect	LEIT	<2.0%	<2.2%	0.2–320 mg/l
Testosterone	Waters^*3^ Acquity UPLC System	LC-MS/MS	<7.1%	<10.9%	0.1–2,000 nmol/l
Cortisol	Waters Acquity UPLC System	LC-MS/MS	<5.6%	<12.9%	0.1–2,000 nmol/l

*DHEA-S, Dehydroepiandrosterone-sulfate; SHBG, sex hormone binding globuin; BCG, bromocresol green method; CLIA, chemiluminescent immunoassay; LC-MS/MS, liquid chromatography tandem mass spectrometry; LEIT, latex-enhanced immunoturbidimetric assay*.

*1 *Siemens Healthineers, Erlangen, Germany*.

*2 *Abbott, Wiesbaden, Germany*.

*3 *Waters, Milford, USA*.

To consider the biologically active fraction of testosterone and for reasons of comparability with previous studies, free testosterone (FT) levels were calculated according to Vermeulen et al. (25).

### Analysis of CAG-Repeat Lengths

DNA was extracted from EDTA blood samples using FlexiGene (Qiagen, Hilden, Germany) according to standard procedures. PCR primers (forward: gccgccgtccaagacctaccgag; reverse: cggctgtgaaggttgctgttcc) were designed flanking the poly-glutamine region downstream Lysine at position 57 in exon 1 of the *AR* (NM_000044.6). The resulting PCR products were purified (QIAquick; Qiagen, Hilden, Germany) and sequenced using nested primers (forward: aagtgatccagaacccggg; reverse: ctcatccaggaccaggtagc) and Brilliant Dye 1.1 (NimaGen, Nijmegen, Netherlands) on a 3130 XL Genetic Analyzer (Thermo Fisher Scientific, Waltham, USA) according to the manufacturer's recommendations. The number of CAG repeats was counted independently by two investigators using CodonCode Aligner (CodonCode Cooperation, Centerville, MA, USA) for display.

### Statistical Analysis

Data handling and statistical analyses were performed with SPSS 26.0 (IBM Corp., Armonk, NY, USA) or R (version 4.0.3, (26)). The “PROCESS” macro (Version 3.5) for SPSS was used for Johnson-Neyman analysis, the “partiallyoverlapping” package for R to account for dependencies between subsamples in the comparison of sample characteristics.

*H*_1_ and *H*_2_ were assessed by two-tailed testing, and results were considered significant at *p* < 0.05. Otherwise, it is explicitly stated if results were considered exploratory or corrected for multiple comparisons controlling the two-tailed false-discovery-rate (FDR) at *q* < 0.05.

Effect size calculations relied on squared semipartial correlations (*sr*^2^) that were converted to Cohen's *d* according to Borenstein et al. (27) for ease of interpretation (small ≥ 0.2, medium ≥ 0.5, large ≥ 0.8) or partial η^2^ = 0.04 (small ≥ 0.01, medium ≥ 0.06, large ≥ 0.14).

### Bivariate Correlations

Bivariate correlations were determined considering the scale of measure as indicated (interval scaled variables: Pearson correlation *r* or Kendall's τ in the presence of outliers identified by box plots; interval scaled and dichotomous variable: point-biserial correlation *r*_*pb*_), separately for the sample of adolescents with a BDI-II score ≤ 13, above 13, and with a confirmed MDD diagnosis. Normality of the variables subjected to correlation analysis was assessed by the Kolmogorov-Smirnov test. The analysis of bivariate correlations was considered exploratory.

### Relationship Between Depression Severity, Free Testosterone, and CAG-RL

Two multiple regression models were specified. The first model included BDI-II score (dependent variable) and FT, CAG-RL, depression status (BDI-II score ≤ 13 and > 13), and all their potential interaction terms (independent variables). All analyses concerning interaction terms were corrected for multiple comparisons, except for those addressing *H*_1_ and *H*_2_. FT and CAG-RL were centered to avoid multicollinearity with their interaction terms, depression status was dummy coded (0 = BDI-II score > 13, 1 = BDI-II score ≤ 13). In addition to the variables included in the first model, the second model also accounted for covariates as suggested by previous studies [age, z-standardized BMI (28), smoking (29)], psychotropic drugs (including antidepressants, neuroleptics, and psychostimulant medication) (30), androstenedione, dehydroepiandrosterone-sulfate (DHEA-S) (15), and cortisol (14). Moreover, serum 25(OH)-vitamin D levels were considered as 44.9% (53/118) of patients were 25(OH)-vitamin D deficient.

PROCESS was employed to perform a Johnson-Neyman analysis. A Johnson-Neyman analysis allows determining for which region or regions of the full range of the CAG-RL distribution there is a significant interaction with FT regarding BDI-II scores. This is achieved by extra- and interpolation based on the assessment of the conditional linear relationship between FT and BDI-II scores given a defined but varying number of CAG repeats (31).

Based on the multiple regression analysis outlined above, results were verified by considering only those patients with a diagnosis of MDD confirmed either by the K-SADS-PL or clinical assessment. As this sensitivity analysis was deemed exploratory, results were not corrected for multiple comparisons.

Normality of the residuals of the multiple regression was assessed by the Kolmogorov-Smirnov test. Heteroscedasticity was considered by applying a heteroscedasticity-consistent standard error estimator for regression coefficients according to Huber and White (HC0). Autocorrelations of residuals were excluded by the Durbin-Watson test, outlier detection relied on Cook's distance (>0.3), and multicollinearity was assessed by variance inflation factors (>5).

## Results

### Descriptives

**Figure 1:**
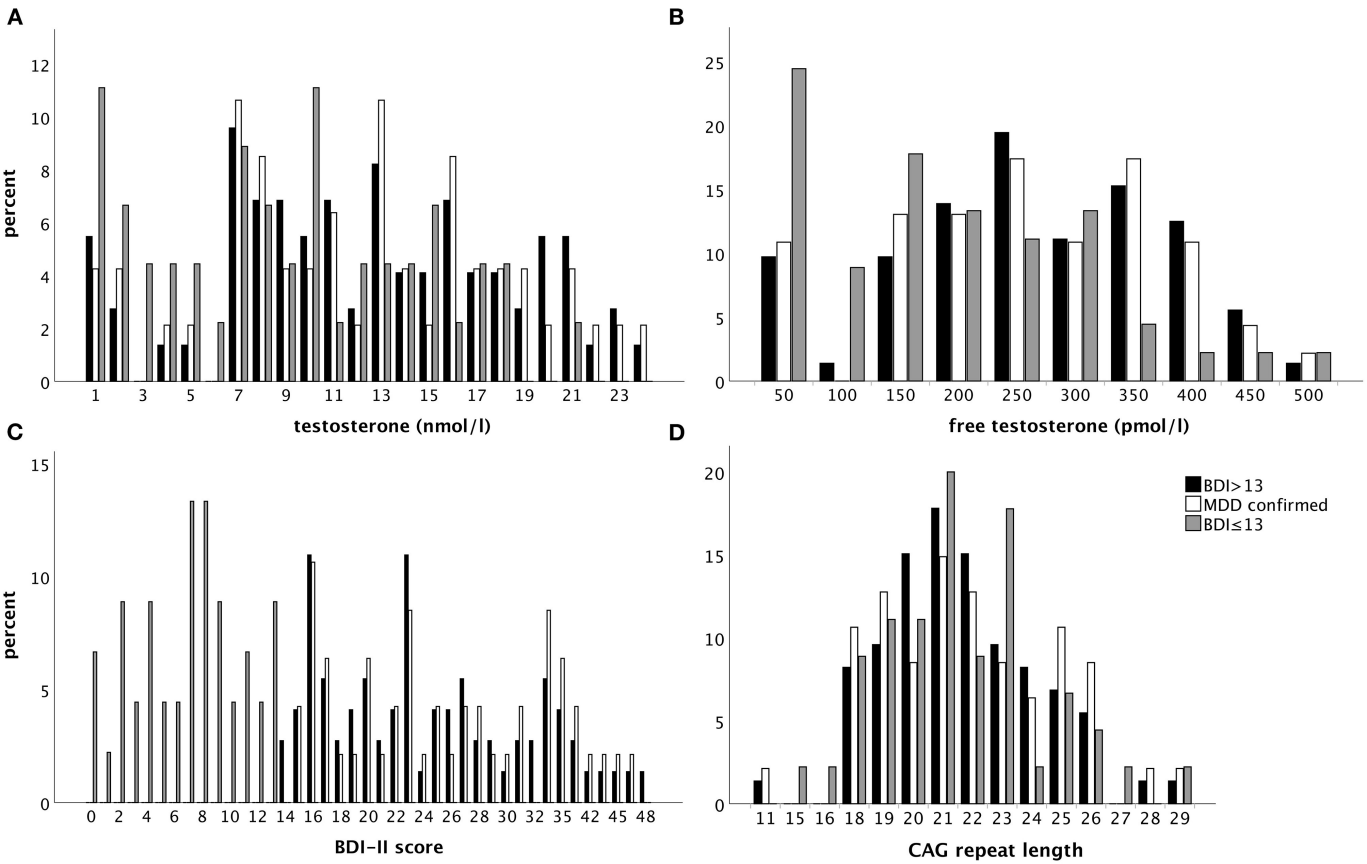
Percentage of patients with regard to the respective sample with a BDI-II score > 13 (black bars), a diagnosis of MDD confirmed by clinical assessment (white bars), or a BDI-II score ≤ 13 (gray bars) considering **(A)** testosterone and **(B)** free testosterone levels as well as **(C)** BDI-II score and **(D)** the CAG repeat length (from left to right and upper to lower panel).

Of the 91 adolescent boys with a BDI-II score > 13, 73 were considered for further analyses. Eleven cases were excluded due to missing information on at least one of the variables of interest, and seven patients due to a BMI below the 5th percentile. These boys had a mean age of 15.70 (SD 1.76) years, were moderately depressed (mean 24.97, SD 8.27), and about 22% (16/73) were taking psychotropic medication, including antidepressants (Table 2, Figure 1). In 4 boys (5.4%), testosterone levels were below 1.0 nmol/l, likely indicating a prepubertal status. The distribution of CAG-RL (9–29 repeats, mean 21.62, SD 2.77) was found to be within the previously reported range in primarily Caucasian populations (9–37 CAG repeats) (13) and did not differ from an earlier study in children and adolescents [(13), mean 21.76, SD 3.06].

**Table 2A:** Patient characteristics.

	**BDI > 13 (*n* = 73)**	**BDI ≤ 13 (*n* = 45)**
Age (years)	15.73 (1.76) [11.80 – 18.39]	14.08 (1.98)[11.26 – 18.38]
**Age distribution (%)**		
11–13 years	11.0	33.3
13–16 years	37.0	44.4
16–18 years	52.0	22.2
z-BMI	0.46 (1.20) [−1.54 – 2.76]	0.48 (1.11) [−1.58 – 2.85]
BDI-II	24.97 (8.27) [14 – 48]	6.84 (3.82) [0 – 13]
**BDI-II severity category (%)**		
Mild	30.1	^#^
Moderate	41.1	^#^
Severe	28.8	^#^
MDD diagnosis - confirmed (%)	64.6	^#^
Psychotropic medication (%)	21.9	17.0
Smoking (%)^*^	30.1	13.2
Migration background (%)	19.2	8.9
Testosterone > 1 nmol/l (%)	94.5	90.6
CAG-RL	21.62 (2.77) [11 – 29]	21.47 (2.84) [15 – 29]
**CAG-RL distribution**		
10. Percentile	18	18
25. Percentile	20	19
50. Percentile	21	22
75. Percentile	23	24
90. Percentile	25	26

*Mean, standard deviation (in round brackets), and range (in square brackets) for interval scaled variables, percentages otherwise; z-BMI, z-standardized BMI; MDD, Major Depressive Disorder; FT, free testosterone; CAG-RL, CAG repeat length*.

* *Significant difference between both groups (P_uncorrected_ < 0.05)*.

# *Does not apply*.

Altogether, there were 64 adolescent boys with a BDI-II score ≤ 13 of which 4 were excluded due to a BMI below the 5th percentile. Another 15 of these patients were excluded due to either incomplete data regarding the variables of interest in the present study (*n* = 7) or an MDD diagnosis (K-SADS-PL or clinical assessment according to ICD-10) despite a BDI-II score ≤ 13 (*n* = 8). This resulted in 45 adolescents with a BDI-II score ≤ 13 with a mean age of 14.08 years (SD 1.98). In 5 patients, biochemical assessment of FT levels indicated a likely prepubertal status. Altogether, 17.8% (8/45) were on psychotropic medication. The average CAG-RL was 21.47 (SD 2.84). The 5 most common diagnoses in patients with a BDI-II score ≤ 13 were: emotional and/or conduct disorder (26.7%, 12/45), attention-deficit hyperactivity disorder (24.4%, 11/45), substance use and addictive disorder (22.2%, 10/45), phobia (13.3%, 6/45), and other behavioral or emotional disorder (8.9%, 4/45). While 12 of the 45 patients (26.7%) had 2 diagnoses, only a single patient (2.2%) had 3 diagnoses.

Adolescents with a BDI-II score ≤ 13 were significantly younger than adolescents with a BDI-II score > 13 (*t*_116_ = −4.72, *P* < 0.001). In line with this, age-dependent hormone levels including FT (*t*_116_ = −3.75, *P* < 0.001) and androstenedione (*U* = 899, *P* < 0.001) were significantly lower in the former, and there was a trend toward lower cortisol (*t*_116_ = −1.89, *P* = 0.06) and DHEA-S levels (*t*_116_ = −1.98, *P* = 0.05) in patients with a BDI-II score ≤ 13 (Table 2B). Moreover, there were significantly fewer current smokers among adolescents with a BDI-II score ≤ 13 than in the sample of adolescents with a BDI-II score > 13 (χ^2^_1_ = 4.34, *P* = 0.04).

**Table 2B:** Selected hormone levels.

	**BDI > 13 (*n* = 73)**	**BDI ≤ 13 (*n* = 45)**
FT (pmol/l)^*^	245.74 (120.53) [0.13–517.61]	160.65 (118.11) [0.01–465.63]
11–13 years	106.31 (107.96) [0–281]	70.73 (77.06) [0–230]
13–16 years	219.59 (98.60) [1–390]	184.59 (119.72) [1–466]
16–18 years	293.67 (110.34) [18–518]	247.65 (74.47) [126–374]
DHEA-S (μmol/l)	6.65 (3.83) [0.08–21.84]	5.49 (2.99) [1.46–15.77]
11–13 years	3.76 (1.33) [1.65–5.18]	3.47 (1.85) [1.45–7.75]
13–16 years	6.08 (4.33) [0.08–21.84]	5.64 (2.12) [2.32–12.12]
16–18 years	8.03 (3.61) [0.08–17.44]	8.21 (3.72) [2.51–15.77]
Androstenedione (nmol/l)^*^	8.67 (5.59) [1.05–34.92]	5.98 (6.37) [1.05–38.41]
11–13 years	3.86 (2.76) [1.05–9.39]	5.15 (9.34) [1.05–38.41]
13–16 years	7.90 (5.15) [1.89–23.36]	5.86 (4.42) [1.29–21.86]
16–18 years	10.23 (5.73) [2.20–34.92]	7.48 (4.26) [3.46–17.84]
Cortisol (nmol/l)	399.36 (133.26) [26.11–665.34]	350.16 (142.97) [150.85–641.75]
11–13 years	253.68 (142.27) [26.11–470.27]	257.85 (122.58) [150.85–602.69]
13–16 years	395.08 (140.33) [162.48–665.34]	369.22 (133.59) [208.18–641.75]
16–18 years	433.07 (105.44) [183.35–615.95]	450.53 (112.66) [231.08–611.27]

*Mean, standard deviation (in round brackets), and range (in square brackets). FT, free testosterone; DHEA-S, Dehydroepiandrosterone-sulfate*.

* *Significant difference between both groups, irrespective of age (p_uncorrected_ < 0.05)*.

There was no significant difference in any of the sample characteristics between adolescents with a BDI-II score > 13 and a clinically diagnosed MDD (*n* = 47; Supplementary Table 1). In the latter group, a diagnosis of MDD was established by the K-SADS-PL in 94.1% (44/47) and *via* clinical assessment according to the ICD-10 in 5.9% (3/47) of cases when no K-SADS-PL was performed on admission.

### BDI-II – Reliability Analysis

Cronbach's alpha was determined to assess the internal consistency of the BDI-II that was found to be excellent (0.91) and in line with previous studies addressing the reliability of the BDI-II in German children and adolescents in a clinical sample (18).

### Bivariate Correlations

**Figure 2:**
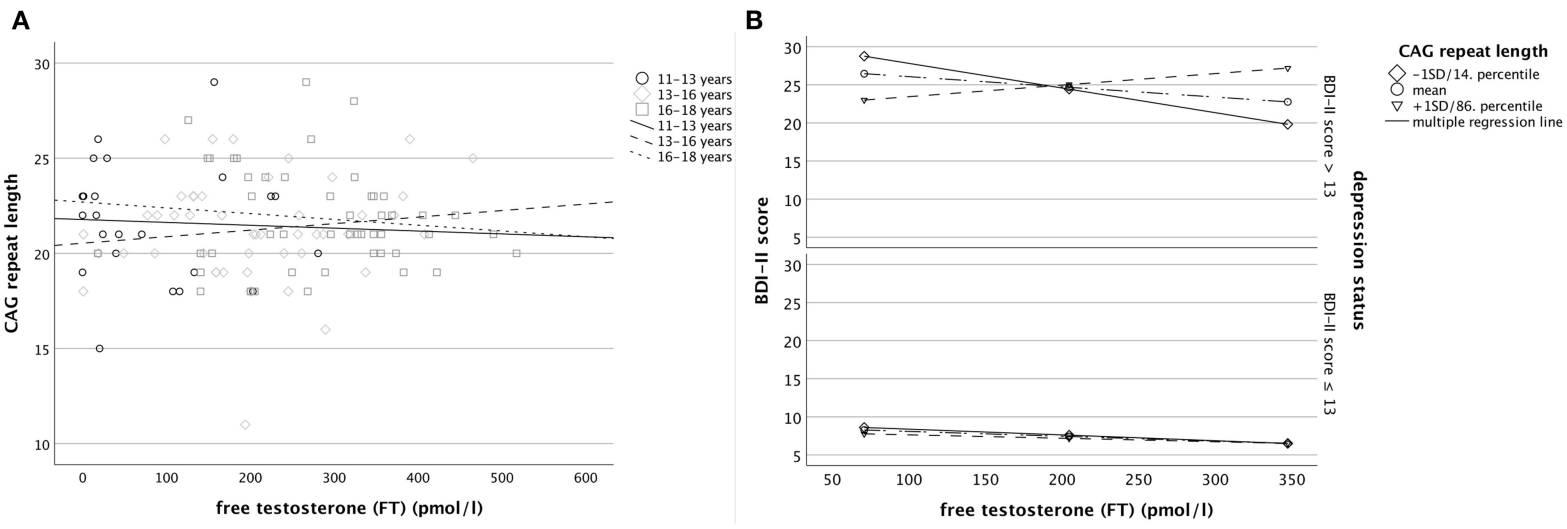
Separately for patients with a BDI-II score > and ≤13, **(A)** the bivariate correlation between free testosterone and the CAG repeat length (left panel) by 3 different age groups and **(B)** regression lines derived from the Johnson-Neyman analysis regarding the linear relationship between free testosterone levels and BDI-II scores individually plotted for exemplary groups of CAG-length [at −1 SD (14. percentile), the mean, and +1 SD (86. percentile); right panel], considering multiple covariates according to model 2.

In adolescents with depression, there was neither a bivariate correlation between BDI-II score and FT (*r*_71_ = 0.04, *P* = 0.73) nor BDI-II score and CAG-RL (*r*_71_ = 0.07, *P* = 0.57; Table 3A). Moreover, there was no correlation between CAG-RL and FT (*r*_71_ = 0.02, *P* = 0.90), which would be expected with a certain relative degree of androgen insensitivity previously reported with an increasing number of CAG repeats of the *AR* (32) (Figure 2A). This also applied when accounting for effects of age (*b* = 0.002, *t*_69_ = 0.65, *P* = 0.517).

**Table 3:** Bivariate correlations.

	**BDI-II score**	**FT**	**CAG-RL**	**DHEA-S**	**Androstenedione**	**Cortisol**	**25(OH)-vitamin D**	**Age**	**z-BMI**	**Smoking**	**Psychotropic medication**
**A. Adolescents with a BDI-II** **>** **13**											
BDI-II score	1	0.04	0.07	0.13	0.05	−0.22	−0.01	0.10	−0.01	0.27^*^	0.00
FT		1	0.02	0.25^*^	0.36^**^	0.35^**^	−0.01	0.54^**^	−0.24^*^	0.39^**^	−0.04
CAG-RL			1	−0.22	−0.01	0.20	0.03	−0.03	0.03	0.22	0.04
DHEA-S				1	0.40^**^	0.16	0.06	0.41^**^	0.17	0.24	−0.19
Androstenedione					1	0.46^**^	0.05	0.34^**^	−0.04	0.26^**^	−0.12
Cortisol						1	0.13	0.36^**^	−0.10	0.21	−0.06
25(OH)-vitamin D							1	−0.09	−0.5	0.11	−0.02
Age								1	−0.06	0.35^*^	0.10
z-BMI									1	−0.13	−0.04
Smoking										1	−0.13
Psychotropic medication											1
**B. Adolescents with a BDI-II** **≤** **13**											
BDI-II score	1	0.12	−0.07	0.01	0.13	0.16	−0.17	0.14	−0.25	0.00	−0.01
FT		1	0.00	0.41^**^	0.54^**^	0.46^**^	−0.22	0.63^**^	−0.17	0.40^**^	−0.07
CAG-RL			1	0.15	0.06	0.17	0.12	0.22	−10	−0.21	−0.02
DHEA-S				1	0.42^**^	0.55^**^	−0.13	0.70^**^	0.07	0.24	−0.17
Androstenedione					1	0.46^**^	−0.11	0.45^**^	0.00	0.25^*^	−0.14
Cortisol						1	−0.11	0.62^**^	0.10	0.24	−0.31^*^
25(OH)-vitamin D							1	−0.26	−0.18	−0.06	0.00
Age								1	−0.12	0.33^**^	−0.05
z-BMI									1	−0.10	−0.30^*^
Smoking										1	−0.01
Psychotropic medication											1

*FT, free testosterone; CAG-RL, CAG repeat length; DHEA-S, Dehydroepiandrosterone-sulfate; z, z-standardized*.

* *P < 0.05*.

** *P < 0.01*.

The same pattern of correlations regarding BDI-II score, FT, and CAG-RL was also observed in patients with a BDI-II score ≤ 13 and patients with a confirmed MDD (Table 3B; Supplementary Table 2).

### Multiple Regression Analysis – Total Sample

Concerning depression severity, there was a three-way interaction between FT, CAG-RL, and depression status (model 1: *b* = −0.01, *t*_110_ = −2.78, *P* = 0.006; *d* = 0.26, Table 4) in line with *H*_2_, and this held upon consideration of multiple covariates (model 2: *b* = −0.01, *t*_101_ = −2.15, *P* = 0.03; *d* = 0.21). More specifically, as revealed by *post-hoc* Johnson-Neyman analysis, the relationship between FT and depression severity was found to be constant across all levels of CAG-RL in adolescents with a BDI-II score ≤ 13 (model 1: *b* = −0.0001, *t*_110_ = −0.07, *p* = 0.95; model 2: *b* = 0.001, *t*_101_ = 0.36, *P* = 0.72; Figure 2B) but to vary as function of CAG-RL in adolescents with a BDI-II score > 13 (model 1: *b* = 0.01, *t*_110_ = 3.11, *P* = 0.002; *d* = 0.39; model 2: *b* = 0.01, *t*_101_ = 2.69, *P* = 0.008; *d* = 0.33). In line with *H*_1_, in the latter, there was a significant negative relationship between FT and BDI-II score with <19 CAG repeats (*b* = −0.03, *t*_101_ = −2.00, *P* = 0.048, partial η^2^ = 0.04) and a significant positive relationship regarding FT and BDI-II score with more than 28 CAG repeats (*b* = 0.06, *t*_101_ = 2.00, *P* = 0.048, partial η^2^ = 0.04), even when considering multiple covariates (model 2). No such relationship was found in patients with an intermediate number of CAG repeats (19-28 CAG repeats).

**Table 4:** Results of the multiple regression analysis.

		**Model 1**						**Model 2**		
**Variables**	***b***	**SE**	***t*** **-value**	***P***	***P***_**FDR-corrected**_	***b***	**SE**	***t*** **-value**	***P***	***P***_**FDR-corrected**_
Age	#	#	#	#	#	0.34	0.54	0.63	0.53	0.83
z-BMI	#	#	#	#	#	−0.28	0.54	−0.70	0.49	0.83
Psychotropic medication	#	#	#	#	#	−1.06	1.55	−0.69	0.49	0.83
Smoking	#	#	#	#	#	3.35	2.11	1.59	0.12	0.42
DHEA-S	#	#	#	#	#	0.06	0.21	0.29	0.77	0.83
Androstendione	#	#	#	#	#	0.23	0.11	2.22	0.03	0.13
Cortisol	#	#	#	#	#	−0.01	0.01	−2.23	0.03	0.13
25(OH)-vitamin D	#	#	#	#	#	−0.02	0.03	−0.66	0.51	0.83
FT	0.02	0.008	0.22	0.83	0.83	−0.01	0.01	−0.77	0.45	0.83
CAG-RL	0.12	0.32	0.38	0.71	0.83	0.20	0.31	0.65	0.52	0.83
Depression status	−17.81	1.13	−15.69	<0.001	<0.001	−17.34	1.13	−15.42	<0.001	<0.001
FT × CAG-RL	0.01	0.003	3.11	0.002	#	0.01	0.004	2.69	0.008	#
FT × group	0.002	0.01	0.22	0.82	0.83	0.002	0.01	0.25	0.81	0.83
CAG-RL × group	−0.22	0.40	−0.55	0.59	0.83	−0.28	0.42	−0.68	0.50	0.83
FT × CAG-RL × group	−0.01	0.004	−2.78	0.006	#	−0.01	0.004	−2.15	0.03	#

*Dependent variable: BDI-II score. b, unstandardized regression coefficient; SE, standard error; z, z-standardized; DHEA-S, Dehydroepiandrosterone-sulfate; FT, free testosterone; CAG-RL, CAG repeat length; group, dummy coded depression status according to the BDI-II score (>13 = 0; ≤13 = 1)*.

# *Does not apply*.

### Multiple Regression Analysis – Sensitivity Analysis

The finding of a significant interaction between FT and CAG-RL was confirmed by sensitivity analysis considering only those patients with a confirmed MDD (model 1: *b* = 0.011, *t*_43_ = 2.61, *P* = 0.01; *d* = 0.79; Supplementary Table 3), also when accounting for multiple covariates (model 2: *b* = 0.01, *t*_35_ = 2.44, *P* = 0.02; *d* = 0.68).

## Discussion

In 73 adolescent boys with depression defined *via* a BDI-II score > 13, there was a significant interaction between FT and the number of CAG repeats of the *AR* regarding depression severity. As indicated by Johnson-Neyman analysis, higher FT levels were associated with a higher BDI-II score when CAG repeats exceeded 28 copies, and a lower BDI-II score when CAG repeats fell below 19 copies, even when controlling for multiple covariates. In an intermediate range of CAG repeats, no such relationship was observed. These findings held up in a subsample of adolescents in whom the diagnosis of depression was verified by clinical assessment. In contrast, in a sample of 45 adolescent boys with a BDI-II score ≤ 13, the relationship between FT and depression severity was found to be independent of CAG-RL.

The finding that with <19 CAG repeats, higher FT levels were associated with less severe depressive symptoms in patients with a BDI-II score >13 is in line with results from two previous studies conducted in depressed adults (11, 12). However, it conflicts with results from a community sample of children and adolescents. In the latter study, fewer CAG repeats and higher FT levels were weakly but significantly related to, in most part, subclinical depression severity (13). Therefore, the relationship between testosterone, CAG-RL, and depression may depend on the severity of the depression (subclinical vs. overt depression), not only regarding its direction but also its size. In line with this, in the present study, no such relationship was found in adolescents with a BDI-II score ≤ 13, and this also applied to older men (≥65) with mostly subclinical depression (92.5%) in a previous community study (33). Considering a multifactorial and threshold model of depression (34), the effect of the interplay between testosterone and CAG-RL on mood may only become apparent once the balance between protective and risk factors has been tipped to develop overt depression. This explanation, however, is tentative and in need of empirical support.

The observation of an opposite relationship between FT and depression severity dependent on the CAG-RL in patients with a BDI-II score > 13 is embedded in an emerging pathogenetic framework. While previous *in vitro* as well as *in vivo* studies indicated that the activity of the androgen receptor is inversely related to the number of CAG repeats of the *AR* (7, 35), this assumption has more recently been challenged. Not only the cell and tissue type examined (36, 37) but also confounding methodological factors substantially modify this relationship (35). As recently summarized and discussed, only CAG-RLs outside the medium range of their distribution are likely implicated in disease pathogenesis and phenotype (35). Moreover, CAG repeats of different lengths affect not only the activity of the androgen receptor but also the set of genes expressed (38). The finding that only those CAG-RLs located (more) closely to the margins of the CAG-RL distribution are oppositely related to the severity of depression in the present study is well in line with both these observations and may also explain that there was no relationship between depression severity and FT levels in patients with an intermediate number of CAG repeats.

However, the relationship between CAG-RL and androgen receptor functioning has been shown to be tissue-specific (36, 37). While the effect of testosterone on adolescent depression is likely related to its effect on brain morphology and functioning (39), the relationship between CAG-RL and androgen receptor functioning in the central nervous system is not well-investigated. Perrin et al. (40) and Paus et al. (41) found a higher correlation between testosterone levels and white matter volume in male adolescents with fewer (≤22) CAG repeats than in those with longer (>23) CAG repeats. As myelination in the adolescent brain is considered an expression of structural maturation related to the effects of androgens (41), this may indicate that a lower number of CAG repeats is related to increased AR functioning in brain tissue. In adults, however, no correlation between CAG-RL and cortical *AR* mRNA, used as a surrogate parameter for androgen receptor functioning, was observed in a small-scale study of psychiatric patients (42). In summary, the effect of testosterone on androgen receptor activity in relation to the CAG-RL on brain function and its implications in mental health is insufficiently understood.

### Limitations

The exact relationship between testosterone and androgen receptor functioning is not fully understood, probably due to confounding factors, including non-genomic effects, potential epigenetic effects, and modifying genetic polymorphisms, which we were not able to account for in the present study. However, neither of these factors has been implicated in depression and its severity in humans so far.

While this is the first study to investigate the relationship between adolescent depression and FT levels as well as the CAG-RL of the *AR* in a clinical sample, our sample of adolescents with a BDI-II score > 13 was limited to 73 boys. However, considering the overall size of the present study, it was adequately powered for valid statistical conclusions (43).

In line with previous studies addressing the relationship between FT, the CAG-RL, and depression severity [e.g., (11–13)], the analysis of the *AR* was based on whole blood samples. Interestingly, in certain neurodegenerative diseases (44, 45), in colorectal cancer (46) as well as in a transgenic animal models to study Huntington's disease (47), somatic mosaicism of CAG repeat length variation has been found. However, we are not aware that a CAG-RL mosaicism has been described in human blood DNA outside the named, specific diseases and animal disease models. Nevertheless, it cannot be excluded that potential mosaicism of the CAG repeat polymorphisms in human blood DNA may exist and may have influenced our data.

Even though accounting for covariates affecting gonadal functioning, the present study did not consider other covariates known to affect depression severity, including measures of cognitive functioning, socioeconomic status, or (traumatic) life events.

Eventually, the present study was cross-sectional by design, and, therefore, causal inference is limited.

### Implications and Future Research

Considering a multifactorial model in the etiology of depression (34) that allows only for small contributions of a single cause, the interaction between FT, CAG-RL, and depression severity in adolescents with MDD can be considered significant not only by means of statistical criteria but also by criteria defined in psychiatry research for an important clinical effect (48).

In line with previous studies (11, 13), the results of the present study imply that in order to understand the relationship between testosterone levels and depression severity in MDD, the number of CAG repeats needs to be considered as their relationship may be obscured otherwise. This not only seems to apply to adults but also to (peri-)pubertal adolescents with depression.

Moreover, there is considerable interest in precision medicine in psychiatry research to identify individuals at risk and to provide targeted therapy (49, 50), also by means of genetic risk profiling (50, 51). The finding that CAG-RLs at the top and bottom of the common CAG-RL distribution are oppositely related to depression severity in MDD depending on FT levels may not only help to explain the heterogeneity of findings in meta-analyses investigating the effect of testosterone treatment on depressive mood (52) but also to identify those individuals that may benefit most from testosterone treatment.

As mentioned above, the relationship between CAG-RL of the *AR* and testosterone in (different types of) brain tissue is hardly understood. Assuming a pivotal role for the brain in the etiology of mental health disorders (51), this could be further investigated given the results of the present study.

Additionally, the CAG-RL of the *AR* may interact with other genetic polymorphisms, not only on the *AR*, to affect behavior and potentially also the risk for mental health disorders, which has not yet been studied in detail. Regarding aggression and impulsivity in men, however, there is preliminary evidence that also the GGN polymorphism of the *AR* gene may modify the effects of varying CAG-RL (36). Therefore, also this aspect of *AR* functioning merits further research.

## Data Availability Statement

The raw data supporting the conclusions of this article will be made available by the authors, without undue reservation.

## Ethics Statement

The studies involving human participants were reviewed and approved by Ethics Committee of the University of Duisburg-Essen (No. 15-6363-BO). Written informed consent to participate in this study was provided by the participants' legal guardian/next of kin.

## Author Contributions

RH and CG conceptualized the present study. RH analyzed and interpreted the data and wrote the manuscript. CG, MF, LL, AH, JB, and JH designed, undertook the clinical studies, and collected the data. P-MH and AK performed the steroid hormone analyses. All authors participated in scientific discussions, revised the manuscript, and approved of the submitted version.

## Funding

RH was supported by the UMEA Clinical Scientist Program by the Faculty of Medicine of the University of Duisburg-Essen and the German Research Foundation (DFG). The authors also acknowledge support by the Open Access Publication Fund of the University of Duisburg-Essen. The funders had no role in the study design, data collection, and analysis, decision to publish, or preparation of the manuscript.

## Conflict of Interest

The authors declare that the research was conducted in the absence of any commercial or financial relationships that could be construed as a potential conflict of interest.

## Publisher's Note

All claims expressed in this article are solely those of the authors and do not necessarily represent those of their affiliated organizations, or those of the publisher, the editors and the reviewers. Any product that may be evaluated in this article, or claim that may be made by its manufacturer, is not guaranteed or endorsed by the publisher.
