# Supplementary material for: Size Matters: The CAG Repeat Length of the Androgen Receptor Gene, Testosterone, and Male Adolescent Depression Severity
Source: Front Psychiatry. 2021 Oct 20;12:732759. doi: 10.3389/fpsyt.2021.732759 (PMC8564040; doi:10.3389/fpsyt.2021.732759)
Supplement: Supplementary file 1 [file Data_Sheet_1.pdf]

Supplementary Material

Outline

Table S1 – Patient Characteristics

Table S2 – Bivariate Correlations

Table S3 - Multiple Regression Analysis

Table S1. Characteristics of Patients with confirmed MDD

age (years)	15.92 (1.64)<br>[11.80 - 18.39]
z-BMI	0.47 (1.26)<br>[-1.54 - 2.76]
BDI-II	26.21 (8.56)<br>[15 - 46]
BDI-II severity category (%)	
mild	25.5
moderate	38.3
severe	36.2
MDD diagnosis - confirmed (%)	100
psychotropic medication (%)	21.3
smoking (%)	27.7
migration background (%)	19.2
testosterone > 1nmol/l (%)	95.7
FT (pmol/l)	246.55 (125.92)<br>[1.16 - 517.61]
CAG-RL	21.79 (3.25)<br>[11 - 29]
CAG-RL distribution	
10. percentile	18
25. percentile	19
50. percentile	22
75. percentile	24
90. percentile	26
DHEA-S (µmol/l)	6.70 (4.15)<br>[0.08 - 21.84]
androstenedione (nmol/l)	0.09 (0.06)<br>[0.01 - 0.35]
cortisol (nmol/l)	379.60 (128.95)<br>[26.11 - 615.95]
25(OH)-vitamin D (nmol/l)	32.85 (15.83)<br>[13.73 - 86.36]
25(OH)-vitamin D < 12 nmol/l (%)	53.2

Mean, standard deviation (in round brackets), and range (in square brackets) for interval scaled variables, percentages otherwise; z-BMI: z-standardized BMI, MDD: Major Depressive Disorder. FT = free testosterone, CAG-RL = CAG repeat length, DHEA-S = dehydroepiandrosterone-sulfate.

Table S2. Bivariate Correlations

	BDI-II score	FT	CAG-RL	DHEA-S	androstenedione	cortisol	25(OH)-vitamin D	age	z-BMI	smoking	psychotropic medication
**BDI-II score**	1	.03	.02	.20	.15	-.06	.00	.15	-.03	.36*	.00
**FT**		1	.05	.23	.40**	.41**	.09	.51**	-.30*	.32*	.05
**CAG-RL**			1	-.28	.00	.36*	-.11	.01	.04	.22	.16
**DHEA-S**				1	.36**	.06	-.07	.27	.19	.26	-.15
**androstenedione**					1	.45**	.05	.34**	-.10	.34**	-.06
**cortisol**						1	.22	.32*	-.22	.32*	.02
**25(OH)-vitamin D**							1	.01	-.23	.21	.10
**age**								1	-.11	.36*	.27
**z-BMI**									1	-.11	-.04
**smoking**										1	-.09
**psychotropic medication**											1

FT = free testosterone, CAG-RL = CAG repeat length, DHEA-S = dehydroepiandrosterone-sulfate, z = z-standardized. * $P < .05$, ** $P < .01$.

Table S3. Results of the multiple regression analysis

	**model 1**				**model 2**			
variables	b	SE	t-value	P	b	SE	t-value	P
age	#	#	#	#	0.06	0.85	0.07	.95
z-BMI	#	#	#	#	-0.19	0.87	-0.22	.83
psychotropic medication	#	#	#	#	0.05	2.89	0.02	.99
smoking	#	#	#	#	6.94	3.18	2.18	.04
FT	0.002	0.01	0.29	.77	-0.02	0.01	-1.11	.28
CAG-RL	0.46	0.35	1.31	.20	0.43	0.45	0.92	.37
FT x CAG-RL	0.01	0.004	2.83	.006	0.01	0.004	2.44	.02
DHEA-S	#	#	#	#	0.04	0.34	0.12	.91
androstendione	#	#	#	#	48.35	23.46	2.06	.047
cortisol	#	#	#	#	-0.02	0.02	-1.32	.20
25(OH)-vitamin D	#	#	#	#	-0.03	0.08	-0.35	.73

Dependent variable: BDI-II score. b = unstandardized regression coefficient, SE = standard error, z = z-standardized. FT = free testosterone, CAG-RL = CAG repeat length, DHEA-S = dehydroepiandrosterone-sulfate. # Does not apply.
